# Application and Performance of Artificial Intelligence (AI) in Oral Cancer Diagnosis and Prediction Using Histopathological Images: A Systematic Review

**DOI:** 10.3390/biomedicines11061612

**Published:** 2023-06-01

**Authors:** Sanjeev B. Khanagar, Lubna Alkadi, Maryam A. Alghilan, Sara Kalagi, Mohammed Awawdeh, Lalitytha Kumar Bijai, Satish Vishwanathaiah, Ali Aldhebaib, Oinam Gokulchandra Singh

**Affiliations:** 1 Preventive Dental Science Department, College of Dentistry, King Saud bin Abdulaziz University for Health Sciences, Riyadh 11426, Saudi Arabia; 2 King Abdullah International Medical Research Centre, Ministry of National Guard Health Affairs, Riyadh 11481, Saudi Arabia; 3 Restorative and Prosthetic Dental Sciences Department, College of Dentistry, King Saud bin Abdulaziz University for Health Sciences, Riyadh 11426, Saudi Arabia; 4 Maxillofacial Surgery and Diagnostic Sciences Department, College of Dentistry, King Saud bin Abdulaziz University for Health Sciences, Riyadh 11426, Saudi Arabia; 5 Department of Preventive Dental Sciences, Division of Pediatric Dentistry, College of Dentistry, Jazan University, Jazan 45142, Saudi Arabia; 6 Radiological Sciences Program, College of Applied Medical Sciences, King Saud bin Abdulaziz University for Health Sciences, Riyadh 11426, Saudi Arabia

**Keywords:** artificial intelligence, histopathological images, oral cancer, diagnosis, cancer classification, cancer prediction, pathology, histopathological features

## Abstract

Oral cancer (OC) is one of the most common forms of head and neck cancer and continues to have the lowest survival rates worldwide, even with advancements in research and therapy. The prognosis of OC has not significantly improved in recent years, presenting a persistent challenge in the biomedical field. In the field of oncology, artificial intelligence (AI) has seen rapid development, with notable successes being reported in recent times. This systematic review aimed to critically appraise the available evidence regarding the utilization of AI in the diagnosis, classification, and prediction of oral cancer (OC) using histopathological images. An electronic search of several databases, including PubMed, Scopus, Embase, the Cochrane Library, Web of Science, Google Scholar, and the Saudi Digital Library, was conducted for articles published between January 2000 and January 2023. Nineteen articles that met the inclusion criteria were then subjected to critical analysis utilizing QUADAS-2, and the certainty of the evidence was assessed using the GRADE approach. AI models have been widely applied in diagnosing oral cancer, differentiating normal and malignant regions, predicting the survival of OC patients, and grading OC. The AI models used in these studies displayed an accuracy in a range from 89.47% to 100%, sensitivity from 97.76% to 99.26%, and specificity ranging from 92% to 99.42%. The models’ abilities to diagnose, classify, and predict the occurrence of OC outperform existing clinical approaches. This demonstrates the potential for AI to deliver a superior level of precision and accuracy, helping pathologists significantly improve their diagnostic outcomes and reduce the probability of errors. Considering these advantages, regulatory bodies and policymakers should expedite the process of approval and marketing of these products for application in clinical scenarios.

## 1. Introduction

Oral cancer (OC) is one of the most common forms of head and neck cancer and continues to have the lowest survival rates worldwide, despite advancements in research and therapy. The prognosis of OC has not significantly improved in recent years, creating a persistent challenge in the biomedical field [1,2]. Cancers of the lip, mouth, and oropharynx, collectively known as oral cancer, are the 13th most prevalent cancer globally [3]. According to the World Health Organization, it is estimated that there were 377,713 new cases and 177,757 deaths from cancers of the lip and oral cavity worldwide in 2020 [3,4]. It is estimated that over 90% of all oral cancers are oral squamous cell carcinomas (OSCCs), which are highly aggressive and have a strong propensity to spread both locally and to other parts of the body [4,5,6]. 

Early detection of OSCC is vital for successful therapy, increased chances of survival, and lower rates of mortality and morbidity [7]. Microscopy-based histopathological analysis of tissue samples is considered the gold standard for diagnosing and grading oral cancer. However, this approach can be slow and prone to errors, limiting its clinical usefulness [8]. Furthermore, it may lead to subjective discrepancies in interpretation and variability in results. This, in turn, may impact the treatment process [9]. Therefore, there is a need for alternative diagnostic methods that can offer greater accuracy, speed, and standardization to overcome these drawbacks. In recent times, significant efforts have been invested in investigating the potential of artificial intelligence (AI) in improving medical diagnosis. Landini and Othman pioneered and developed an automated method that utilizes morphological reconstruction to report the architectural characteristics of the epithelium for oral cancer diagnosis [10]. Additionally, digital pathology is gaining traction in quantitative analysis as an effective approach by leveraging high-performance computer technology [11,12,13].

Machine learning (ML) techniques identify distinguishable patterns from existing data but rely on human knowledge and efforts to distinguish features. Deep learning (DL), a subset of machine learning that employs artificial neural networks to imitate the human brain’s process of learning, is a recent advancement that can directly extract features from raw images. Both ML and DL algorithms improve the performance of computer-aided diagnostic systems (CAD) with more training samples [14]. As a result, researchers have integrated image processing, pattern recognition, machine learning, and deep learning methods to develop CAD for oral cancer diagnosis. The literature suggests that automated quantification of an oral cancer diagnosis reduces grading conflicts between pathologists [15].

The major advantage of AI is that it reduces the load of manual visualization of slides. It also assists pathologists in fast decision making with better accuracy. Computerized image analysis of tissue slides can obtain information that may be missed with traditional viewing of slides. Precise and accurate histological findings are necessary for early diagnosis, classification, prediction, and specific treatment planning for OC [16]. Various reports have been published describing the application of AI in the early diagnosis, prognosis, and classification of OC [4,5,6,13,14,17,18,19]. This systematic review is exclusively intended to evaluate the performance of AI in oral cancer detection, diagnosis, classification, and prediction using histopathological images.

## 2. Materials and Methods

### 2.1. Search Strategy

The authors of this systematic review followed the diagnostic test accuracy guidelines set forth in the Preferred Reporting Items for Systematic Reviews and Meta-Analyses Extension (PRISMA-DTA) [20] to ensure methodological quality. The search was conducted based on the PICO (Problem/Patient, Intervention/Indicator, Comparison, and Outcome) criteria, which are presented in Table 1. 

An electronic search was conducted using several reputable databases, including PubMed, Scopus, Embase, the Cochrane Library, Web of Science, Google Scholar, and the Saudi Digital Library, for articles published between January 2000 and January 2023. The index words used for the search of the articles were artificial intelligence, automated models, histopathology, histopathological images, slides, hematoxylin and eosin-stained images, oral pathology, biopsy, computational pathology, oral cancer diagnosis, oral cancer detection, oral cancer classification, oral cancer prognosis, cancer cell detection, epithelial layer, keratin pearl, keratinization, artificial neural networks (ANN), supervised learning, unsupervised learning, machine learning, and deep learning. The article search was performed in electronic databases utilizing Boolean operators (AND, OR), along with a filter for years (2000–2023) and a language filter for English. In addition to our electronic search, we also conducted a manual search for relevant research articles and citations. This involved reviewing the reference lists of previously retrieved articles in the college’s library. The search was performed independently by two qualified authors.

### 2.2. Study Selection

A total of 590 articles were obtained through the electronic database search, and an additional four articles were retrieved through the manual search, resulting in a total of 594 articles for initial consideration. The initial selection of articles was based on their relevance to the research area, as well as the title and abstract. To ensure that there were no duplicated articles, two members not involved in the initial search checked all articles for duplicates, leading to the removal of 322 duplicates. Subsequently, 272 full-text articles were thoroughly reviewed for data selection, with eligibility criteria being applied at this stage.

### 2.3. Inclusion and Exclusion Criteria

The inclusion criteria for selecting articles were as follows: (a) the article must be original research and must report on AI technology, (b) the article should mention quantifiable values that can be evaluated/analyzed, and (c) articles should mention the data used to evaluate the AI-based models. The study design was not restricted for inclusion in this systematic review. On the other hand, the following types of articles were excluded: (a) those that did not mention AI innovation, (b) unpublished articles or conference papers uploaded online, (c) articles that did not have full-text versions available, and (d) articles available in languages other than English. 

### 2.4. Data Extraction

**Figure 1:**
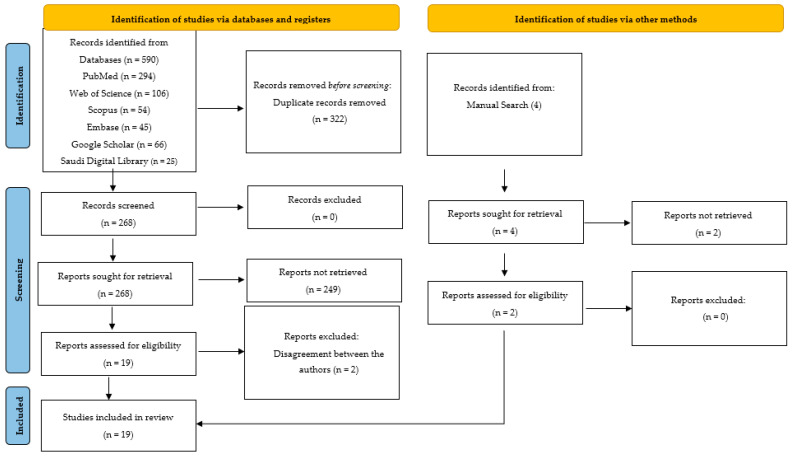
PRISMA 2020 flow diagram for new systematic reviews that included searches of databases, registers, and other sources.

Following the application of the inclusion criteria, 21 articles were initially selected for analysis. In the second phase, the journal and author details were removed from the articles, and two independent authors who were not involved in the initial search (S.B.K. and L.A.) evaluated them critically. The data from the selected articles were extracted and entered into a Microsoft Excel sheet. These data included publication year, study objectives, AI algorithm types used, and data utilized for training, validation, and testing of the model, as well as the results, conclusions, and recommendations made. However, due to insufficient data to substantiate the results and conclusions of two articles, there were contrasting opinions among the authors regarding their inclusion. After discussing the matter with another expert author (M.A.), a decision was made to exclude them. Therefore, a total of 19 articles were ultimately included for quantitative synthesis, as illustrated in Figure 1. These 19 articles were considered to be potentially eligible articles for this systematic review and were critically analyzed.

A quality assessment of the included articles was carried out using QUADAS-2. It comprises four domains that assess different aspects of study design and reporting, including patient selection, index test, reference standard, and flow and timing [21]. The reliability between the two reviewers was tested using Cohen’s kappa on a sample of articles, displaying an 88% level of agreement. By evaluating each domain for risk of bias and applicability concerns, researchers can identify potential sources of bias and assess the generalizability of the results to different clinical settings and patient populations. 

## 3. Results

After conducting a critical analysis of the 19 articles, qualitative data were extracted. The majority of the studies, which were reported over the past seven years, revealed an increasing trend of reporting on the use of AI for OC diagnosis and prognostic prediction using histopathological images.

### 3.1. Qualitative Synthesis of the Included Studies

AI technology has been mainly applied for diagnosing OSCC [22,23,24,25,26,27,28], differentiating between normal and malignant conditions [29,30,31,32,33,34,35,36], forming an early diagnosis of OSCC [37], predicting the survival of patients [38,39], and grading the severity of OSCC [40]. In this systematic review, 17 studies were reported using convolutional neural networks (CNNs), while the other two were conducted utilizing capsule networks and a hybrid technique (CNN + ANN), as depicted in Table 2 [15,16].

### 3.2. Study Characteristics 

The extracted features from the studies included information on the authors, publication year, study objectives, AI model development algorithm type, data sources utilized for model training, validation and testing, evaluation accuracy, conclusions, and recommendations.

### 3.3. Outcome Measures 

Task performance efficiency was assessed using various outcome measures, including accuracy, sensitivity, specificity, precision, recall, receiver operating characteristic curve (ROC), area under the curve (AUC), statistical significance, F1 scores, positive predictive value (PPV), negative predictive value (NPV), discrete wavelet transform (DWT), local binary pattern (LBP), fuzzy color histogram (FCH), gray level co-occurrence matrix (GLCM), mean intersection-over-union (mIOU), Dice coefficient, and Jaccard Index.

### 3.4. Risk of Bias Assessment and Applicability Concern

**Figure 2:**
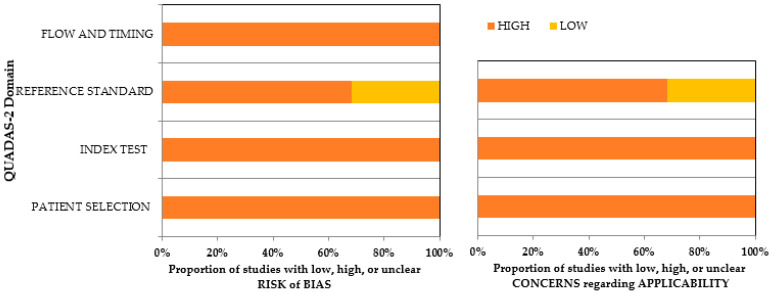
QUADAS-2 assessment of the individual risk of bias domains and applicability concerns.

The quality and risk of bias of the included studies were assessed using the QUADAS-2 assessment tool (Table S1). All studies utilized histopathological images as input for the neural networks, resulting in a low risk of bias for the patient-selection domain in both arms. Standardized techniques were used for data feeding in AI technology, leading to a low risk of bias for flow and timing. All the studies had implemented a highly uniform training system, resulting in a low risk of bias for the index test in both arms of QUADAS-2. Six studies [22,23,25,28,38,39] had used human observations as the reference standard, and hence 30% of the studies reported a high risk in bias assessment and applicability concern. Overall, there was a low risk of bias in both arms across all categories of the included studies. Details regarding the risk of bias assessment and the applicability of the included studies are provided in Supplementary Table S1 and Figure 2.

### 3.5. Assessment of the Strength of Evidence 

The Grading of Recommendations Assessment Development and Evaluation (GRADE) approach was utilized to determine the certainty of the evidence in this systematic review [41]. The certainty of evidence was evaluated based on five domains: risk of bias, inconsistency, indirectness, imprecision, and publication bias. It is classified as very low, low, moderate, or high certainty of evidence. Based on this assessment, the overall included studies in this systematic review demonstrated high certainty of evidence (Table 3).

## 4. Discussion

The healthcare industry is experiencing the rising power and potential of AI innovations in enhancing the quality of clinical care. AI technologies have the capacity to assist clinicians in minimizing human errors and achieving more precise decision making with superior outcomes than traditional approaches [42]. Deep CNN represents a promising advancement in AI that utilizes algorithms based on neural networks that imitate human neuron mechanisms. CNNs are currently under development as tools to support clinicians in solving various challenges and improving the accuracy of disease detection in radiographic and clinical images [43]. It is important to note that AI technology is not intended to replace clinicians but to aid them in making more precise evaluations and diagnoses of patients [44].

The field of head and neck cancer diagnosis has seen a rapid influx of AI applications that have shown promising results in the preliminary interpretation of medical images [45]. Detecting tumoral changes early on is crucial to ensure timely surgical intervention, subsequent treatment, and, ultimately, increased survival rates. Additionally, this can significantly lower postsurgical morbidity rates and improve quality of life, particularly in cases of invasive and malignant tumors [46].

From its inception, OC is a disease that is often aggressive and resistant to treatment in its more advanced stages. A five-year outlook can vary significantly, with early-stage detection providing an 84% survival rate, while late detection in stages III and IV drops to a 39% survival rate [47]. Additionally, postoperative quality of life declines significantly, particularly for those in advanced stages [48]. 

The identification of OSCC demands rigorous histopathological investigation, which requires tissue preparation and consumes a considerable amount of time. Moreover, in the case of extensive tumors, multiple samples from different sites need to be removed, and surgeons have to scrutinize the excised margins repeatedly to ensure a clear and cancer-free space [7]. However, prompt and reliable histopathological assessment may not be feasible under certain circumstances [8]. Hence, the implementation of AI as an auxiliary screening tool presents itself as a significant opportunity to enhance diagnostic accuracy noninvasively. In this systematic review, we endeavored to appraise the efficacy of AI in the detection, diagnosis, classification, and prediction of OC from histopathological images [46].

### 4.1. Effectiveness of AI in the Diagnosis of Oral Cancer 

Advanced machine learning algorithms are revolutionizing the field of oncology, particularly in the diagnosis of oral cancer. These models offer a swift and noninvasive method of detecting lesions at a level of accuracy that rivals leading human specialists [49]. While the oral cavity is readily accessible during routine check-ups, many cancers often go unnoticed until they reach advanced stages. Thus, the use of AI offers the potential for a solution to combat the high mortality rates associated with OC [50,51]. Of the pool of articles analyzed in our study, a total of seven studies considered the possibility of utilizing AI-based models for diagnosing OC. One noteworthy study is by Das et al., in which they developed a segmentation method that could identify keratin pearls and quantify the keratinization layer in the oral mucosa of patients with OC. The study utilized a keratinization index (CKI) measure for the automated diagnosis and grading of OSCC. The results were promising, demonstrating the potential for using AI to diagnose oral cancer through quantitative analysis of microscopic images of oral tissues at lower magnification [22].

Hameed et al. introduced a novel technique leveraging the power of machine learning to score immunohistochemistry (IHC) [23]. This involves identifying the most tightly linked feature elements, thereby automating the scoring process. The accuracy of this methodology was compared against manual IHC scores provided by two observers, which were then statistically evaluated. The experiment revealed that the automated IHC score, generated from the top 10 most interdependent feature elements out of a total of 214, has a high correlation coefficient (CC) to the manual scores provided by the observers. Therefore, this finding confirms that the proposed automated IHC scoring mechanism has promising potential in the analysis of IHC-stained tissues [23].

Deif et al. achieved a superior standard of diagnostic efficiency in OSCC patients using histopathological images, employing Inception V3 with BPSO to attain a classification accuracy of 96.3%. This approach not only enhanced accuracy but also effectively curtailed diagnostic costs. While the formidable performance of deep learning algorithms is undeniable, the authors stressed the need for further research to corroborate their efficacy on larger datasets and to compare their results to those of human experts [24].

In a recent study by Yang et al., a deep learning algorithm outperformed pathologists in accurately identifying OSCC in medical images. Moreover, when aided by an AI model, junior pathologists were able to identify OSCC in images 6.26 min faster than when working alone. The model improved the F1 score for both junior (0.922 to 0.957) and senior pathologists (0.936 to 0.946), indicating its potential for improving the accuracy of diagnoses. However, it is important to note that the algorithm was only trained and tested on images from a single institution, and further evaluations are required to determine its generalizability to other populations [25].

In a study by Das et al., a novel two-stage approach was devised to develop improved techniques for the processing of oral histology images. The first stage entails the utilization of a deep CNN comprising 12 convoluted layers of 7 × 7 × 3 channel patches that collate and segment the constituent layers. In the subsequent stage, they detect keratin pearls in the segmented keratin regions by harnessing the power of texture-based features through a Gabor filter-trained random forest. Through this pioneering methodology, they achieved a detection accuracy of 96.88% for the identification of keratin pearls [26].

The second study, conducted by Das et al. in 2019, focused on the development of a computer-aided tool for detecting and delineating nuclei from oral histopathology images to aid in OSCC screening. The authors utilized a combination of texture analysis and machine learning techniques to create an algorithm that could automatically segment nuclei from histological images. The deep learning algorithm underwent training and testing on images from a single institution, potentially limiting the generalizability of its outcomes to other populations [27].

Yoshizawa et al. devised a method using automated machine learning to distinguish OSCC cases based on the YK classification through digital images obtained from histopathological specimens. The method produced strong outcomes overall, with an F-value of 0.87. However, the authors were unable to employ H&E-stained images, even though doing so would be cost-effective and pragmatic. Deep learning could elevate classification accuracy, but acquiring an adequate number of cases remains a crucial roadblock. To augment the precision of classification via deep learning, an ample number of samples would be mandatory. Regrettably, they were unable to obtain the requisite number of cases to achieve this [28].

### 4.2. Effectiveness of AI in Differentiating Normal from Malignant Regions

Rahman et al. categorized histological slides of oral squamous cell carcinoma into normal (benign) or abnormal (malignant) based on microscopic images. Texture features of the images were analyzed using GLCM, and histogram techniques were used for feature extraction. Linear SVM was used for classification, resulting in 100% accuracy and satisfactory outcomes [29].

Martino et al. carried out a study to evaluate the segmentation performance of four deep networks using the mean intersection-over-union (mIOU) metric. The findings revealed that the U-Net modified with ResNet50 as an encoder performed better than the original U-Net due to its deeper structure. The authors also highlighted the potential of using an automated segmentation algorithm for oral squamous cell carcinoma [30].

According to the research conducted by Das et al., the proposed CNN exhibited superior performance in comparison to other methods in the form of the highest accuracy, precision, and recall metrics. The average accuracy of the pre-trained VGG-19 and Resnet-50 models exceeded 80%, while Alexnet and VGG-16 exhibited the poorest outcomes in terms of accuracy, precision, and recall. [31].

Fraz et al. proposed a deep learning network called Fabnet for simultaneous segmentation of microvessels and nerves in commonly used H&E-stained histology images. The study showed promising results, suggesting that Fabnet can accurately delineate microvessels and nerves, even in challenging cases, outperforming other semantic segmentation networks in terms of accuracy. This achievement may potentially reduce processing time by only segmenting the identified regions of interest. Therefore, Fabnet potentially paves the way for more efficient segmentation of histology images with microvessels and nerves [32].

The system proposed by Rahman et al. demonstrated a high level of accuracy in identifying unknown classes using color, texture, and shape features. Specifically, the system achieved 100% accuracy with color features and high accuracies of 99.4% and 100% with shape and texture features, respectively. This method offers the advantage of accurate classification and computational efficiency, making it a useful tool for automated oral cancer diagnosis or as an assistive tool for physicians to validate their findings [33].

The concatenated model developed by Amin et al. improved the performance for identifying both cancerous and normal images. Similarly, a high AUC value of 0.997 demonstrates that the concatenated model is highly capable of differentiating between the two classes [34]. 

Panigrahi et al. reported on classifying and grading oral histopathological images through ResNet architecture’s various forms and depths. They obtained optimal results with less computational complexity and a small dataset by using the ResNet13-A as a computer-aided automated model [35].

In another study, Panigrahi et al. used capsule networks (CapsNets), which represent novel machine learning architectures that aim to improve the modeling of hierarchical pose relationships. This is achieved through the use of capsules, which can be defined as collections of neurons that represent an object’s instantiation parameters, such as its pose and orientation. To enable effective routing of the capsule vectors in successive layers, a dynamic routing algorithm is employed. The result is a part-to-whole relationship that is not present in conventional CNNs. CapsNets have been shown to outperform CNNs on the same datasets, demonstrating their enhanced classification capabilities. Additionally, as CapsNets can handle spatial data, they provide better accuracy (97.35%) compared to CNNs (96.77%). The loss function of CapsNets is approximately 0.083 when evaluated on test datasets, while the validation loss of CNNs is 0.132 [36]. This learning curve can be used to evaluate and select the appropriate classifier for a given dataset [36].

### 4.3. Effectiveness of AI in Early Diagnosis of OC

In the realm of histological analysis for oral cancer detection, researchers have sought to enhance the diagnostic process by employing a hybrid approach. Fati et al. explored two innovative methodologies involving a combination of machine learning techniques, such as CNN, support vector machines (SVM), and ANN [37].

In their first approach, they implemented a two-part hybrid method utilizing the power of CNN models, such as AlexNet and ResNet-18, to extract deep features. These features underwent PCA algorithmic intervention to minimize dimensionality. SVM algorithms were tagged in the second phase of diagnosis to accurately classify these features depicting higher ratings. The researchers observed promising outcomes when they utilized this methodology to diagnose the OSCC dataset.

The second approach adopted the use of ANN techniques, which were grounded on hybrid features obtained by integrating color, texture, and shape features derived from other algorithms, such as discrete wavelet transform (DWT), local binary pattern (LBP), fuzzy color histogram (FCH), and gray level co-occurrence matrix (GLCM). This approach effectively diagnosed histological images of oral cancer cells, targeting early detection with admirable diagnosis rates. By harnessing the hybrid features of the ResNet-18, DWT, LBP, FCH, and GLCM algorithms, the ANN methodology achieved 99.3% accuracy, 99.42% specificity, 99.26% sensitivity, and 99.31% precision, with a 99.39% AUC [37]. The results attained by these novel techniques could greatly improve the current diagnostic capabilities of specialists, aiding diagnostic decision making.

### 4.4. Effectiveness of AI in Predicting Survival of OC Patients

Traditional statistical techniques, such as the Cox proportional hazard, have been employed to predict the survival of OC patients; however, they prove to be inadequate when it comes to such intricate conditions. A complex “dataset” for oral carcinoma necessitates an AI-based predictive system to yield promising results [17].

Lu et al. developed a classifier based on image analysis that uses quantitative histomorphometric features to assess nuclear shape, size, and texture diversity in clusters of cells from 2 mm OSCC microarray tumor sections that have been digitized using H&E slides. However, this study has some limitations. The image analysis was conducted only on tissue microarrays, which represent a small portion of the complete tumor. Therefore, it may not capture all the morphological variations that exist in the same tumor. The use of whole-slide images may provide a more complete picture of tumors. Additionally, the study’s sample size was relatively small, and some of the well-established clinical and pathological features, such as depth of invasion and nodal extracapsular extension, were not controlled for. A larger, statistically powered retrospective cohort of patients should be analyzed to validate the classifier’s effectiveness on whole-slide images while controlling for all of the established clinical and pathologic features, as well as within well-established patient outcome subgroups [38]. 

The digital score for TIL abundance was calculated to investigate its potential as a prognostic marker for DFS in OSCC patients in a study conducted by Shaban et al. The TIL abundance score (TILAb) was computed based on the classification of tumor and lymphocytic regions. State-of-the-art CNN-based image classifiers were employed for tissue region classification. The classifiers with the highest (TRC-5) and lowest (TRC-1) patch-level classification accuracy were chosen for further analysis of TIL detection, score computation, and survival analysis. Significant results were obtained by both classifiers [39].

### 4.5. Effectiveness of AI in the Grading of OC

An innovative method to grade oral tumors using fuzzy cognitive maps (FCM) was developed by Anuradha et al. The FCM model uses eight histopathological features, and an active Hebbian learning (AHL) algorithm is utilized as the supervised learning mechanism to train and improve the model’s grading system. To test the accuracy of the FCM and AHL approach, 123 cases, including 85 normal and 38 abnormal oral tumors, were assessed. The model achieved an accuracy of 90.58% for low-grade oral tumors and 89.47% for high-grade tumors, demonstrating its potential as an important tool in the effective diagnosis and grading of oral tumors. This innovative approach has the potential to improve patient outcomes and reduce morbidity and mortality rates associated with oral tumors [40].

## 5. Future Perspectives and Limitations

The field of oncology has made significant progress with the incorporation of deep learning algorithms. These intelligent systems assist pathologists in effectively classifying cancer across multiple categories, thereby empowering the oncology team to chart out a treatment module, reducing the operational workload and enhancing disease management. Moreover, deep learning models allow clinicians to classify patients into different risk categories for determining the most suitable treatment [31]. This approach could spare those who do not fall into the high-risk bracket from the more unpleasant side effects of intensive treatments. However, while this has the potential to pave the way for AI to be widely implemented, data privacy and confidentiality remain obstacles in applying AI to clinical oncology [52,53]. Of particular concerns are the potential interpretation errors that could arise while relying heavily on software for medical diagnoses and who should bear ultimate responsibility—the digital intelligence or the skilled doctor [18]. Additionally, AI’s introduction into oncology practice has the potential to impact the patient–doctor relationship and the patient’s autonomy. AI models are designed to assist pathologists and clinicians in clinical decision making. These models have demonstrated outstanding results in performing these tasks. However, when there is discordance between the AI models and the human experts, the latter make the final decision based on their clinical expertise [54]. AI models developed for application in histopathological diagnosis are based on ML and DL, which are subsets of AI. DL models are easy to use in comparison to ML models and have better accuracy, as they are suitable for large sets of data. Moreover, the input of the defined features is not required, as their performance continues to improve with more practice [55]. DL models have an added advantage due to their ability to work on unstructured data and to generate new features with higher quality from datasets without human interventions, which improves their accuracy in diagnosis [56]. 

One of the limitations of these AI applications is the problem of the interpretability and explainability of the operation of these algorithms. AI models should provide clinicians and patients with a complete understanding of their decisions. However, to date, there has been no unified method for evaluating interpretability [57]. All of these concerns require careful consideration in order to arrive at an appropriate solution.

## 6. Conclusions

This systematic review provides evidence in support of machine learning models and their significant potential for delivering highly accurate detection of OC with better sensitivity, specificity, and precision. This can significantly aid pathologists in improving their diagnostic outcomes and reducing the probability of errors. Artificial intelligence presents remarkable prospects for the automation of tasks by identifying intricate patterns. Therefore, it is imperative to investigate and promote the integration of AI techniques across disciplines. Such advancements could pave the way for further exploration and research in the future. The diligent scrutiny and surveillance of AI systems to ensure their security, efficiency, and equitability are of critical importance. The growing interest in the development of these advanced models requires an assessment of their quality and application to ensure their safety and cost-effectiveness before being deployed in clinical scenarios. However, considering the advantages, regulatory bodies and policymakers should expedite the process of approval and marketing of these products for application in clinical practice. 

## Figures and Tables

**Table 1:** Description of the PICO (P = Population, I = Intervention, C = Comparison, O = Outcome) elements.

Research Question	What is the Performance of the Artificial Intelligence Models That Have Been Widely Used in Oral Cancer Detection, Diagnosis, Classification, and Prediction Using Histopathological Images?
Population	Patients who underwent investigations for oral cancer (histological images).
Intervention	AI-based models designed for oral cancer diagnosis, classification, and prediction of prognosis.
Comparison	Expert opinions and reference standards.
Outcome	Measurable or predictive outcomes, such as accuracy, sensitivity, specificity, precision, recall, receiver operating characteristic curve (ROC), area under the curve (AUC), statistical significance, F1 scores, positive predictive value (PPV), negative predictive value (NPV), discrete wavelet transform (DWT), local binary pattern (LBP), fuzzy color histogram (FCH), gray level co-occurrence matrix (GLCM), mean intersection-over-union (mIOU), Dice coefficient, and Jaccard index.

**Table 2:** Details of the studies that have used AI-based models in oral cancer detection, diagnosis, classification, and prediction using histopathological images.

Sl No	Authors	Year of Publication	Study Design	Algorithm Architecture	Objective of the Study	No. of Images/Photographs for Testing	Study Factor	Modality	Comparison if any	Evaluation Accuracy/Average Accuracy/Statistical Significance	Results (+) Effective, (−) Non-Effective (N) Neutral	Outcomes	Authors’ Suggestions/Conclusions
1	Das DK et al. [22]	2015	Observational study	CNN	To develop a computer-assisted quantitative microscopic methodology for automated identification of keratinization and keratin pearl areas from in situ oral histological images	10 OSCC patients’ oral histological slides	Diagnosis of OSCC	Histopathological images	Manual experts	95.08% segmentation accuracy	(+) Effective	This provides a computer-aided diagnostic framework for microscopic image-based OSCC diagnosis, which can assist clinicians/pathologists for rapid evaluation.	The proposed methodology would be able to provide more robust performance when the sample size is large and can also be recommended as one of the tele-pathology applications.
2	Hameed KA et al. [23]	2016	Observational study	CNN	To develop automatic IHC scoring of p53-immunostained tissue images of oral cancer	400 sub-regions of tissue images	Diagnosis of OSCC	Histopathological images	State-of-the-art methods, including intensity and texture features (manual)	Classification accuracy of 96.09% achieved by the proposed method for LDA classifiers	(+) Effective	The automatic scoring methods presented have high potential in IHC image analysis.	It helps the pathologist during the diagnostic and prognostic evaluation of oral cancer.
3	Deif MA et al. [24]	2022	Observational study	CNN	To diagnose OSCC using deep neural networks	Histopathological images of 230 individuals	Diagnosis of OSCC	Histopathological images	VGG16, AlexNet, ResNet50, and Inception V3	Accuracy of 96.3% was obtained when using Inception V3 with BPSO.	(+) Effective	Best classification accuracy of 96.3% was obtained when using Inception V3 with BPSO.	This approach significantly contributes to improve the diagnostic efficiency of OCSCC patients while reducing diagnostic costs.
4	Yang SY et al. [25]	2022	Observational study	CNN	To develop a custom-made deep learning model to assist pathologists in detecting OSCC from histopathology images	2025 images	Diagnosis of OSCC	Histopathological images	3 junior pathologists, 3 senior pathologists, and 1 chief pathologist	Sensitivity of 0.98, specificity of 0.92, positive predictive value of 0.924, negative predictive value of 0.978, and F1 score of 0.951	(+) Effective	The results demonstrated that the automated deep learning method could evaluate OSCC approximately 249 times faster than a junior pathologist.	These findings indicate that deep learning can improve the accuracy and speed of OSCC diagnosis from histopathology images.
5	Das DK et al. [26]	2018	Observational study	CNN	To identify clinically relevant regions from oral tissue histological images for OSCC diagnosis	42 tissue slides	Computer-aided diagnosis and screening of oral cancers	Histopathological images	Gabor filter-trained random forest	Epithelial layer segmentation: 98.42% segmentation accuracy, 97.76% sensitivity, 90.63% Jaccard index, and 95.03% Dice coefficient. Keratin pearls: 98.05% segmentation accuracy, 71.87% Jaccard index, and 75.19% Dice coefficient.	(+) Effective	Proposed approach is good enough to extract epithelial, subepithelial, and keratin regions from oral histological images.	Segmentation of epithelial and subepithelial layers and detection of keratin pearls can be utilized for oral precancerous screening and OSCC grading, respectively.
6	Das DK et al. [27]	2019	Observational study	CNN	To develop a two-stage computational pipeline for automatic detection of nucleus and its segmentation from oral histology images	42 tissue slides	Computer-aided diagnosis of OSCC	Histopathological images	Chan–Vese model	94.22% Dice coefficient, 89.38% Jaccard index, 88.87% recall, and 82.03% precision	(+) Effective	The proposed segmentation methodology performed well, with 94.22% Dice coefficient, 89.38% Jaccard index, 97.56% precision, and 91.58% recall.	This is the first attempt on oral tissue histology image computation for joint nucleus detection and segmentation to diagnose OSCC.
7	Yoshizawa K et al. [28]	2022	Observational study	CNN	To determine the mode of invasion based on digital images of the invasive front of an OSCC.	101 digitized photographic images	Diagnosis of OSCC	Histopathological images	Yamamoto–Kohama grades (1, 2, 3, 4C, 4D) determined by a human oral and maxillofacial surgeon	F-measure value of 87%	(+) Effective	These results suggest that the output of the classifier was very similar to the judgments of the clinician.	This system may be valuable for diagnostic support to provide an accurate determination of the mode of invasion.
8	Rahman TY et al. [29]	2019	Observational study	CNN	To develop a CAD system for OSCC classification using textural features on real histopathologic images	134 images with normal tissue and 135 images with malignant tissue	Differentiating normal and malignant	Histopathological images	Linear support vector machine (SVM)	100% accuracy AUC = 0.92	(+) Effective	The linear support vector machine classifier provided 100% accuracy for the automated diagnosis of oral cancer.	It can be used to assist clinicians in the rapid evaluation and differentiation of tumorous lesions and normal tissue.
9	Martino F et al. [30]	2020	Observational study	SSNs	To compare four different deep learning-based architectures for oral cancer segmentation	188 images	Differentiating normal and malignant areas	Histopathological images	SegNet, U-Net, U-Net with VGG16 encoder, and U-Net with ResNet50 encoder	mIOU SegNet = 0.54 U-Net = 0.57 U-Net with VGG16 encoder = 0.62 U-Net with ResNet50 encoder = 0.63	(+) Effective	The deeper network, U-Net modified with ResNet50 as the encoder, performed better than the original U-Net (having a more shallow encoder).	This will help those who work in generalist diagnostic centres, not specialized in the diagnosis of an infrequent but extremely lethal disease.
10	Das N et al. [31]	2020	Observational study	CNN	To classify OSCC into its four classes as per Broder’s system of histological grading	156 slide images	Differentiation of malignant lesions in biopsy images	Histopathological images	Alexnet, Resnet-50, VGG 16, and VGG 19	Accuracy of 97.5%.	(+) Effective	Highest classification accuracy of 92.15% was achieved with the Resnet-50 model. The proposed CNN model outperformed the transfer learning approaches, displaying an accuracy of 97.5%.	It can be concluded that the proposed CNN-based multi-class grading method of OSCC could be used for the diagnosis of patients with OSCC.
11	Fraz MM et al. [32]	2020	Observational study	CNN	To propose a deep network for simultaneous segmentation of microvessels and nerves in routinely used H&E-stained histology images	7780 images	Differentiating normal from malignant areas	Histopathological images	FCN-8, U-Net, Segnet, and DeepLabv3	Accuracies of 96.3% and 97.05% for nerves and blood vessels	(+) Effective	The proposed network outperformed the current deep neural networks used for semantic segmentation.	The proposed network also provides robust segmentation performance when applied to the full digital whole slide image.
12	Rahman TY et al. [33]	2021	Observational study	CNN	To propose an automated efficient computer-aided system to distinguish normal from malignant OSCC categories	42 slides	Classification of cell nuclei into normal and malignant categories	Histopathological images	Decision tree classifier, SVM, and logistic regression	99.4% accuracy using decision tree classifier, 100% accuracy using both SVM and logistic regression, and 100% accuracy using SVM	(+) Effective	The in-depth analysis showed SVM and linear discriminant classifiers provided the best results for texture and color features, respectively.	This system is fast, cost-effective, and accurate. Hence, physicians can use it in their daily clinical screening as an assistant diagnostic tool.
13	Amin I et al. [34]	2021	Observational study	CNN	To propose an automated classification of cancerous oral histo pathological images	290 normal and 934 cancerous oral histo pathological images	Differentiating normal and malignant areas	Histopathological images	VGG16, InceptionV3, and Resnet50	96.66% accuracy, 95.16% precision, 98.33% recall, and 95.00% specificity; concatenated model AUC = 0.997	(+) Effective	The concatenated model yielded the best results and outperformed the individual models.	These results demonstrate that the concatenated model can effectively replace the use of a single DL architecture.
14	Panigrahi S et al. [35]	2022	Observational study	CNN	To propose three ResNet architectures for the multistage classification of OSCC into benign and malignant	400 image patches	Differentiating normal from malignant	Histopathological images	ResNet-based model	97.59% accuracy	(+) Effective	The Optimal ResNet model (ResNet13-A) was chosen as the best model, which is an automated computer-aided method to obtain high-performance results with less computational complexity and small datasets.	The proposed ResNet model is an efficient model for detecting multistage oral cancer, and it can be utilized as a diagnostic tool to help physicians in daily clinical screening.
15	Panigrahi S et al. [36]	2022	Observational study	Capsule network	To classify oral cancer using a deep learning technique known as capsule network to discriminate between cancerous and non-cancerous images	82 malignant and 68 benign images	Differentiating normal and malignant areas	Histopathological images	Regular CNN model	97.78% sensitivity, 96.92% specificity, and 97.35% accuracy	(+) Effective	Capsule networks have better capabilities in capturing the pose information and spatial relationship and can better discriminate between cancerous and non-cancerous images compared to the CNN model.	The proposed system can be extended to classify the different stages of oral cancer in the future.
16	Fati SM et al. [37]	2022	Observational study	CNN ANN	To achieve satisfactory results for the early diagnosis of OSCC by applying hybrid techniques based on fused features	5192 images	Early diagnosis of OSCC	Histopathological images	CNN models (AlexNet and ResNet-18) and SVM algorithm ANN models (ResNet-18, DWT, LBP, FCH, and GLCM)	ResNet-18, DWT, LBP, FCH, and GLCM achieved an accuracy of 99.3%, specificity of 99.42%, sensitivity of 99.26%, precision of 99.71%, and AUC of 99.39%	(+) Effective	The ANN algorithm based on hybrid features yielded promising results in histological image diagnostics for early diagnosis of OSCC.	This study highlights the tremendous potential of artificial intelligence techniques to diagnose OSCC and increase cure rates among patients.
17	Lu C et al. [38]	2017	Observational study	CNN	To construct an oral cavity histomorphometric-based image classifier for risk stratification of OSCC patients	Slides from 115 patients	To risk stratify patients for disease-specific survival	Histopathological image	Standard clinical and pathologic parameters	ROC = 0.72, hazard ratio = 11.02	(+) Effective	Patients with positive results were 11 times more likely to develop disease recurrence and die from it.	Quantitative histomorphometric features of local nuclear architecture derived from digitized H&E slides of OSCCs are independently predictive of patient survival
18	Shaban M et al. [39]	2019	Observational study	CNN	To obtain an automated TIL abundance score and explore its prognostic significance for disease-free survival (DFS) of OSCC patients	Slides from 70 patients	Prognostic significance for disease-free survival of OSCC patients	Histopathological images	Manual TIL score	High accuracy of 96.31%	(+) Effective	The automated TILAb score had a significantly higher prognostic value than the manual TIL score (*p* = 0.0024).	The TILAb score can be used as an independent prognostic parameter in OSCC patients.
19	Anuradha K et al. [40]	2017	Observational study	CNN	To histologically grade oral tumors using fuzzy cognitive map (FCM)	Histopathological images from 123 cases	Computer-aided grading of oral tumors	Histopathological images	Active Hebbian learning (AHL)	Accuracy of 90.58% for oral tumors of low grade and 89.47% of high grade	(+) Effective	The proposed method used an FCM grading model to categorize tumor cases into low grade and high grade. In addition, to improve the values, an active Hebbian learning algorithm was used.	Features can be extracted using feature extraction methods and can be given as input to the FCM.

Footnotes: OSCC = oral squamous cell carcinoma, CNNs = convolutional neural networks, SNNs = semantic segmentation deep neural networks, ROC = receiver operating characteristic curve, AUC = area under the curve, IHC = immunohistochemical, TIL = tumor infiltrating lymphocytes, mIOU = mean intersection-over-union, DWT = discrete wavelet transform, LBP = local binary pattern, FCH = fuzzy color histogram, and GLCM = gray level co-occurrence matrix.

**Table 3:** Assessment of Strength of Evidence.

Outcome	Inconsistency	Indirectness	Imprecision	Risk of Bias	Publication Bias	Strength of Evidence
Application of AI in the diagnosis of OSCC [22,23,24,25,26,27,28]	Not Present	Not Present	Not Present	Present	Not Present	⨁⨁⨁◯
Application of AI in differentiating between normal and malignant conditions [29,30,31,32,33,34,35,36]	Not Present	Not Present	Not Present	Not Present	Not Present	⨁⨁⨁⨁
Application of AI in the early diagnosis of OSCC [37]	Not Present	Not Present	Not Present	Not Present	Not Present	⨁⨁⨁⨁
Application of AI in predicting survival of OSCC patients [38,39]	Not Present	Not Present	Not Present	Present	Not Present	⨁⨁⨁◯
Application of AI in severity grading of OSCC [40]	Not Present	Not Present	Not Present	Not Present	Not Present	⨁⨁⨁⨁

Foot Notes: ⨁⨁⨁⨁—high evidence, ⨁⨁⨁◯—moderate evidence.

## Data Availability

Not applicable.

## Supplementary Materials

The following supporting information can be downloaded at https://www.mdpi.com/article/10.3390/biomedicines11061612/s1, Table S1: Assessment of risk of bias domains and applicability concerns.
