# Supplementary material for: Application and Performance of Artificial Intelligence (AI) in Oral Cancer Diagnosis and Prediction Using Histopathological Images: A Systematic Review
Source: Biomedicines. 2023 Jun 1;11(6):1612. doi: 10.3390/biomedicines11061612 (PMC10295336; doi:10.3390/biomedicines11061612)
Supplement: Supplementary file 1 [file biomedicines-11-01612-s001.zip › biomedicines-2396562-supplementary.pdf]

Table S1. Assessment of risk of bias domains and applicability concerns.

		RISK OF BIAS				APPLICABILITY CONCERNS		
Serial no	Authors	Patient Selection	Index Test	Reference Standard	Flow And Timing	Patient Selection	Index Test	Reference Standard
1	Das DK et al.[22]	LOW	LOW	HIGH	LOW	LOW	LOW	HIGH
2	Hameed KA et al.[23]	LOW	LOW	HIGH	LOW	LOW	LOW	HIGH
3	Deif MA et al.[24]	LOW	LOW	LOW	LOW	LOW	LOW	LOW
4	Yang SY et al.[25]	LOW	LOW	HIGH	LOW	LOW	LOW	HIGH
5	Das DK et al.[26]	LOW	LOW	LOW	LOW	LOW	LOW	LOW
6	Das DK et al.[27]	LOW	LOW	LOW	LOW	LOW	LOW	LOW
7	Yoshizawa K et al.[28]	LOW	LOW	HIGH	LOW	LOW	LOW	HIGH
8	Rahman TY et al.[29]	LOW	LOW	LOW	LOW	LOW	LOW	LOW
9	Martino F et al.[30]	LOW	LOW	LOW	LOW	LOW	LOW	LOW
10	Das N et al.[31]	LOW	LOW	LOW	LOW	LOW	LOW	LOW
11	Fraz MM et al.[32]	LOW	LOW	LOW	LOW	LOW	LOW	LOW
12	Rahman TY et al.[33]	LOW	LOW	LOW	LOW	LOW	LOW	LOW
13	Amin I et al.[34]	LOW	LOW	LOW	LOW	LOW	LOW	LOW
14	Panigrahi S et al.[35]	LOW	LOW	LOW	LOW	LOW	LOW	LOW
15	Panigrahi S et al.[36]	LOW	LOW	LOW	LOW	LOW	LOW	LOW
16	Fati SM et al.[37]	LOW	LOW	LOW	LOW	LOW	LOW	LOW
17	Lu C et al.[38]	LOW	LOW	HIGH	LOW	LOW	LOW	HIGH
18	Shaban M et al.[39]	LOW	LOW	HIGH	LOW	LOW	LOW	HIGH
19	Anuradha K et al.[40]	LOW	LOW	LOW	LOW	LOW	LOW	LOW
